# Time trends of surgical treatment and the prognosis for Japanese patients with gastric cancer

**DOI:** 10.1054/bjoc.2000.1427

**Published:** 2000-10

**Authors:** Y Maehara, Y Kakeji, S Oda, I Takahashi, K Akazawa, K Sugimachi

**Affiliations:** Department of Surgery and Science, Graduate School of Medical Sciences, Kyushu University, Fukuoka, Japan; Department of Medical Informatics, Niigata University Medical Hospital, Niigata University, Niigata, Japan

**Keywords:** gastric cancer, surgical treatment, lymph node dissection, recurrence, prognosis

## Abstract

The incidence of gastric cancer is much higher in Japan than in other countries even though diagnostics and treatments of such patients have improved. The objective of this study was to present an overview of the past, present and future of surgical treatment for our patients with gastric cancer. We analysed data on 2152 Japanese men and women with gastric cancer who underwent surgical resection from 1965 to 1995 at Kyushu University in Fukuoka, Japan, based on a univariate and the multivariate analysis. We focused on time trends of surgical treatment and the postoperative outcome. Over the years, there have been favourable changes in the numbers of patients with early gastric cancer. In all cases of gastric cancer, the rate of 18% in the first six year period (group 1) was 57% in the last 5 year period (group 6). Size of the tumour was smaller, well-differentiated tumour tissue was more common, and lymphatic involvement was less frequent. Lymph node metastasis, liver metastasis and peritoneal dissemination all decreased. Extensive lymph node dissection was more frequently done and the rate of curative resection (curability A and B) increased. With increases in identifying the early stage of cancer and better perioperative care, mortality rates 30 days after the surgery greatly decreased. Multivariate analysis revealed that the 10 factors of depth of invasion, lymph node metastasis, lymph node dissection, tumour size, liver metastasis, peritoneal dissemination, lymphatic invasion, vascular invasion, lesion in the whole stomach and lesion in the middle stomach were independent factors for determining the prognosis. Detection of the tumour in an early stage, standardized surgical treatment, including routine lymph node dissection, close follow-up schedules and better perioperative management are expected to increase survival time for patients with this malignancy. © 2000 Cancer Research Campaign


British Journal of Cancer (2000) **83**(8), 986–991
© 2000 Cancer Research Campaign
doi: 10.1054/ bjoc.2000.1427, available online at http://www.idealibrary.com on IDEAL®

### Time trends of surgical treatment and the prognosis for Japanese patients with gastric cancer

**Y Maehara, Y Kakeji, S Oda, I Takahashi, K Akazawa† and K Sugimachi**

Department of Surgery and Science, Graduate School of Medical Sciences, Kyushu University, Fukuoka, Japan †Department of Medical Informatics, Niigata University Medical Hospital, Niigata University, Niigata, Japan

**Summary** The incidence of gastric cancer is much higher in Japan than in other countries even though diagnostics and treatments of such patients have improved. The objective of this study was to present an overview of the past, present and future of surgical treatment for our patients with gastric cancer. We analysed data on 2152 Japanese men and women with gastric cancer who underwent surgical resection from 1965 to 1995 at Kyushu University in Fukuoka, Japan, based on a univariate and the multivariate analysis. We focused on time trends of surgical treatment and the postoperative outcome. Over the years, there have been favourable changes in the numbers of patients with early gastric cancer. In all cases of gastric cancer, the rate of 18% in the first six year period (group 1) was 57% in the last 5 year period (group 6). Size of the tumour was smaller, well-differentiated tumour tissue was more common, and lymphatic involvement was less frequent. Lymph node metastasis, liver metastasis and peritoneal dissemination all decreased. Extensive lymph node dissection was more frequently done and the rate of curative resection (curability A and B) increased. With increases in identifying the early stage of cancer and better perioperative care, mortality rates 30 days after the surgery greatly decreased. Multivariate analysis revealed that the 10 factors of depth of invasion, lymph node metastasis, lymph node dissection, tumour size, liver metastasis, peritoneal dissemination, lymphatic invasion, vascular invasion, lesion in the whole stomach and lesion in the middle stomach were independent factors for determining the prognosis. Detection of the tumour in an early stage, standardized surgical treatment, including routine lymph node dissection, close follow-up schedules and better perioperative management are expected to increase survival time for patients with this malignancy. © 2000 Cancer Research Campaign

**Keywords**: gastric cancer; surgical treatment; lymph node dissection; recurrence; prognosis

Gastric cancer remains one of the most serious health problems in Japan (Moreaux and Bougaran, 1993; Fuch and Mayer, 1995). The overall increase in life span and the development of new diagnostic techniques have led to improved detection of gastric cancer (Maehara et al, 1995a). One reason for this better outcome is the predominance of cases of gastric cancer diagnosed at an early stage (Hisamichi, 1989; Maehara et al, 1993). Mass screening with double contrast barium study and endoscopy with biopsy have coincided with an increased recognition of patients with early gastric cancer, fewer patients with advanced gastric cancer, and a decrease in the number of cancer deaths (Kaneko et al, 1977; Noguchi et al, 1989; Hanazaki et al, 1997). Since the 1960s, radical procedures have been introduced for extensive lymph node dissection and the combined resection of adjacent organs (Korenaga et al, 1988; Maehara et al, 1992, 1996b). In potentially curable patients with a localized tumour and no evidence of distant metastasis, extensive en bloc resection led to a substantial increase in survival time, compared with findings in case of a simple gastrectomy, a standard procedure in Japan in the 1950s (Kodama et al, 1981; Korenaga et al, 1988). More radical surgical techniques have been introduced; and anaesthesia, perioperative care, and nutritional support have all improved (Jentschura et al, 1997). These changes resulted in improvement in the outcome for patients over 70 years of age and with gastric cancer (Maehara et al, 1995a). In Western countries, the rate of early gastric cancer increased in time trends, but was much lower, compared to related events in Japan (Sue-Ling et al, 1993; Jentschura et al, 1997). We find no recent reports on time trends of tumour advance and postoperative prognosis for subjects with gastric cancer, as based on analysis of a large number of patients treated in one institution. We determined time trends of clinicopathologic characteristics and postoperative prognosis for 2152 patients with gastric cancer treated with surgical resection between 1965 and 1995, the objective being to present an overview of the past, present and future of the surgical treatment for patients with gastric cancer, in Japan.

*Received 7 December 1999*
*Revised 29 May 2000*
*Accepted 22 June 2000*

*Correspondence to:* Y Maehara

### PATIENTS AND METHODS

### Participants and surgical treatment

From 1965 to 1995, 2152 Japanese patients with gastric cancer and no evidence of any other malignancy underwent gastric resection in the Department of Surgery II, Kyushu University Hospital, Fukuoka, Japan. Details of patient data have been meticulously managed in those periods. The 2152 patients were grouped into six: group 1 (1965–1970, $n$ = 503); group 2 (1971–1975, $n$ = 350); group 3 (1976–1980, $n$ = 333); group 4 (1981–1985, $n$ = 325); group 5 (1986–1990, $n$ = 293); group 6 (1991–1995, $n$ = 348). Inoperable or non-resected cases (because of advanced stages of tumours) numbered 130 (20.5%, 130/633) for group 1, 41 (11.7%. 41/391) for group 2, 32 (9.6%, 32/365) for group 3, 43 (13.2%, 43/368) for group 4, 32 (9.8%, 32/325) for group 5 and 25 (6.7%, 25/373) for group 6. From 1975 on, the stomach was resected after

**Table 1** Clinicopathological characteristics in serial cohorts of Japanese patients who underwent surgery for gastric cancer

| Factor | | Group 1 (*n* = 503) | Group 2 (*n* = 350) | Group 3 (*n* = 333) | Group 4 (*n* = 325) | Group 5 (*n* = 293) | Group 6 (*n* = 348) | *P* value |
|---|---|---|---|---|---|---|---|---|
| Age(yrs) | | 55.3 ± 11.8* | 57.5 ± 12.3 | 59.2 ± 12.3 | 61.2 ± 12.3 | 61.5 ± 12.5 | 62.0 ± 12.1 | 0.0001 |
| Sex | Male | 349(69.4) | 235 (67.1) | 215 (64.6) | 222 (68.3) | 205 (70.0) | 250 (71.8) | 0.420 |
| | Female | 154 (30.6) | 115 (32.9) | 118 (35.4) | 103 (31.7) | 88 (30.0) | 98 (28.2) | |
| Location of the tumour | Upper | 93 (18.5) | 88 (25.1) | 68 (20.4) | 71 (21.8) | 69 (23.5) | 88 (25.3) | 0.0001 |
| | Middle | 138 (27.4) | 90 (25.7) | 103 (30.9) | 85 (26.2) | 108 (36.9) | 117 (33.6) | |
| | Lower | 223 (44.3) | 143 (40.9) | 113 (33.9) | 138 (42.5) | 99 (33.8) | 130 (37.4) | |
| | Whole stomach | 49 (9.8) | 29 (8.3) | 49 (14.8) | 31 (9.5) | 16 (5.5) | 13 (3.7) | |
| Tumour maximal diameter (cm) | | 6.8±3.6* | 7.0±4.0 | 7.8±4.5 | 6.0±4.0 | 4.9±3.3 | 4.9±4.0 | 0.0001 |
| Histology | | | | | | | | |
| Well differentiated | | 230 (46.4) | 195 (55.7) | 147 (44.5) | 153 (47.1) | 151 (52.1) | 185 (53.3) | 0.0148 |
| Poorly differentiated | | 266 (53.6) | 155 (44.3) | 183 (55.5) | 172 (52.9) | 139 (47.9) | 162 (46.7) | |
| Others** | | 7 | 0 | 3 | 0 | 3 | 1 | |
| Depth of invasion | | | | | | | | |
| m | | 39 (7.7) | 41 (11.7) | 38 (11.4) | 62 (19.1) | 82 (28.0) | 117 (33.6) | 0.0001 |
| sm | | 50 (9.9) | 51 (14.6) | 43 (12.9) | 60 (18.5) | 60 (20.5) | 80 (23.0) | |
| mp | | 50 (9.9) | 27 (7.7) | 31 (9.3) | 22 (6.8) | 19 (6.5) | 25 (7.2) | |
| ss | | 63 (12.5) | 38 (10.9) | 42 (12.6) | 18 (5.5) | 35 (11.9) | 49 (14.1) | |
| se | | 209 (41.6) | 122 (34.9) | 111 (33.3) | 122 (37.5) | 80 (27.3) | 66 (19.0) | |
| si | | 92 (18.3) | 71 (20.3) | 68 (20.4) | 41 (12.6) | 17 (5.8) | 11 (3.2) | |
| Lymphatic involvement | | | | | | | | |
| Negative | | 35 (32.1) | 140 (41.8) | 156 (47.4) | 182 (56.5) | 155 (53.6) | 234 (67.2) | 0.0001 |
| Positive | | 74 (67.9) | 195 (58.2) | 173 (52.6) | 140 (43.5) | 134 (46.4) | 114 (32.8) | |
| Unknown** | | 394 | 15 | 4 | 3 | 4 | 0 | |
| Vascular involvement | | | | | | | | |
| Negative | | 47 (78.3) | 264 (79.8) | 265 (84.1) | 265 (82.3) | 198 (69.7) | 260 (75.1) | 0.0002 |
| Positive | | 13 (21.7) | 67 (20.2) | 50 (15.9) | 57 (17.7) | 86 (30.3) | 86 (24.9) | |
| Unknown** | | 443 | 19 | 18 | 3 | 9 | 2 | |
| Histological lymph node metastasis | | | | | | | | |
| Negative | | 184 (36.7) | 139 (39.8) | 134 (40.2) | 143 (44.0) | 168 (58.3) | 218 (63.4) | 0.0001 |
| Positive | | 318 (63.3) | 210 (60.2) | 199 (59.8) | 182 (56.0) | 120 (41.7) | 126 (36.6) | |
| Unknown** | | 1 | 1 | 0 | 0 | 5 | 4 | |
| Peritoneal dissemination | | | | | | | | |
| Negative | | 448 (89.1) | 309 (88.3) | 290 (87.1) | 308 (94.8) | 282 (96.2) | 337 (96.8) | 0.0001 |
| Positive | | 55 (10.9) | 41 (11.7) | 43 (12.9) | 17 (5.2) | 11 (3.8) | 11 (3.2) | |
| Liver metastasis | | | | | | | | |
| Negative | | 475 (94.4) | 330 (94.3) | 316 (94.9) | 310 (95.4) | 287 (98.0) | 340 (97.7) | 0.0001 |
| Positive | | 28 (5.6) | 20 (5.7) | 17 (5.1) | 15 (4.6) | 6 (2.0) | 8 (2.3) | |
| Stage | | | | | | | | |
| Ia | | 81 (16.1) | 80 (22.8) | 69 (20.8) | 109 (33.5) | 130 (45.1) | 179 (51.9) | 0.0001 |
| Ib | | 54 (10.7) | 37 (10.6) | 46 (13.8) | 21 (6.5) | 31 (10.8) | 37 (10.7) | |
| II | | 70 (13.9) | 37 (10.6) | 36 (10.8) | 43 (13.2) | 28 (9.7) | 39 (11.3) | |
| IIIa | | 69 (13.7) | 42 (12.0) | 42 (12.6) | 40 (12.3) | 37 (12.8) | 36 (10.4) | |
| IIIb | | 77 (15.4) | 50 (14.3) | 42 (12.6) | 46 (14.2) | 27 (9.4) | 18 (5.3) | |
| IVa,b | | 152 (30.2) | 104 (29.7) | 98 (29.4) | 66 (20.3) | 35 (12.2) | 36 (10.4) | |
| Unknown | | 0 | 0 | 0 | 0 | 5 | 3 | |
| Gastric resection | | | | | | | | |
| Partial | | 156 (32.3) | 118 (33.7) | 144 (43.9) | 155 (47.8) | 113 (39.0) | 144 (41.3) | 0.0001 |
| Total | | 326 (67.7) | 232 (66.3) | 184 (56.1) | 169 (52.2) | 177 (61.0) | 204 (58.7) | |
| Unknown* | | 21 | 0 | 5 | 1 | 3 | 0 | |
| Lymph node dissection | | | | | | | | |
| $D_0$ & $D_1$ | | 137 (27.2) | 70 (20.0) | 93 (27.9) | 84 (25.8) | 63 (21.5) | 37 (10.6) | 0.0001 |
| $D_2$ & $D_3$ | | 366 (72.8) | 280 (80.0) | 240 (74.1) | 241 (74.2) | 230 (78.5) | 311 (89.4) | |
| Operative curability | | | | | | | | |
| A | | 155 (30.9) | 132 (37.8) | 127 (38.1) | 148 (45.5) | 172 (59.5) | 237 (68.5) | 0.0001 |
| B | | 171 (34.1) | 109 (31.2) | 98 (29.4) | 93 (28.6) | 70 (24.2) | 66 (19.1) | |
| C | | 176 (35.1) | 108 (30.9) | 108 (32.4) | 84 (25.8) | 47 (16.3) | 43 (12.4) | |
| Unkown* | | 1 | 1 | 0 | 0 | 4 | 2 | |

*, mean ± standard deviation
**, Cases excluded from statistical analysis.
m, mucosa; sm, submucosa; mp, muscularis propria; ss, subserosa; se, serosa; si, infiltration into adjacent organs

*© 2000 Cancer Research Campaign*

determining the resection line at least 3 cm apart from the macroscopic edge of the localized tumour and 6 cm for the infiltrative tumour in case of an advanced tumour, and 2 cm for the localized tumour and 4 cm for the infiltrative lesion in the early tumour (Kawasaki et al, 1975). All patients were examined clinically and pathologically with respect to factors given in Table 1. Pathologic diagnosis and classification of the resected gastric cancer tissues were made according to the General Rules for the Gastric Cancer Study in Surgery and Pathology in Japan (Japanese Gastric Cancer Association, 1998). The lymph nodes in groups 1, 2 and 3 are referred to as n1, n2 and n3, respectively, based on the degree of lymph node metastasis. Lymph node dissection was classified as follows: D1, complete removal of group 1 lymph nodes alone; D2, complete removal of group 1 and 2 lymph nodes; and D3, complete removal of group 1, 2 and 3 lymph nodes. Curability A means no evidence of residual tumours and with a high probability of cure, under the following conditions: no serosal invasion; n0 treated by D1, 2, 3 or n1 treated by D2, 3; M0, P0, H0; and the proximal and distal margins >10 mm. Curability B means no evidence of residual tumours but not evaluable as 'Curability A', and Curability C means definite residual tumour.

After surgery the patients were entered into a regular follow-up programs with each or a combination of blood examinations, roentgenography of the gastrointestinal tract, endoscopic procedures, angiography (from 1970), computed tomography and ultrasonography (from 1980). Physical conditions were examined every 2 weeks in the outpatient department for the first year, and every 1 to 2 months after 2 years. After 5 years, the cause of death was obtained from the local physician and always confirmed by a check of the death certificate.

### Statistical methods

The BMDP Statistical Package program (BMDP; Los Angeles, CA) for the IBM (Armonk, NY) 3090 mainframe computer was used for all analyses (Dixon, 1988). The BMDP 4F and 3S programs were used for chi-square and Kruskal-Wallis tests to compare the data. The BMDP 1L program was used to analyse survival time by the Kaplan-Meier method and the Mantel-Cox test was used to test for equality of the survival curves. The BMDP 2L program for simultaneous multivariate adjustment of all covariates by the Cox regression analysis with forward stepwise model was used to determine independent factors related to the prognosis (Cox, 1972). The level of significance was $P < 0.05$.

### RESULTS

### Clinicopathologic factors

The clinicopathologic characteristics of patients are given in Table 1. Patients of a more advanced age were seen in recent periods and the mean age was 7 years older in group 6, compared to that of group 1. The male to female ratio was about 2:1 for each group. Tumours in the whole stomach were fewer and those in upper locations of the stomach increased in recent periods. Tumour size was smaller, and the peak was noted in case of less than 2 cm, in group 6 (Figure 1). The well-differentiated tissue type was more common compared to the poorly differentiated type in recent periods, and the ratio between well differentiated and poorly differentiated has been reciprocal in time trends. In recent periods, depth of invasion was less prominent, in particular, cases of mucosal and submucosal early gastric cancer significantly increased, and in group 6, 58.6% of the patients had an early gastric cancer. The rates of lymphatic invasion and lymph node metastasis were much lower in recent periods. The rates of vascular invasion did not decrease in time trends. The rates of peritoneal dissemination and liver metastasis were also lower. Thus, tumours seen in recent periods appear to be less advanced and detection rates of early stage of gastric cancer have significantly increased. Stage I was noted in 26.8% of patients for group 1 and 62.5% for group 6.

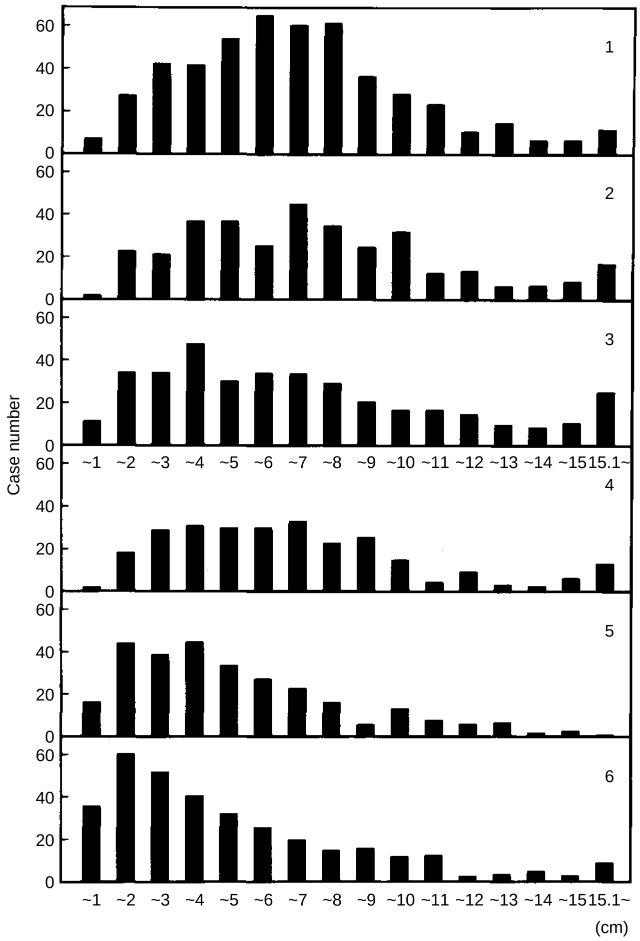

**Figure 1** Tumour size in successive cohorts of patients with gastric cancer. Group 1 (1965–1970, $n$ = 503); group 2 (1971–1975, $n$ = 350); group 3 (1976–1980, $n$ = 333); group 4 (1981–1985, $n$ = 325); group 5 (1986–1990, $n$ = 293); group 6 (1991–1995, $n$ = 348)

### Surgical treatment

As shown in Table 1, the rate of partial gastrectomy increased compared to total gastrectomy because of the dominance of early and small tumours. Extensive lymph node dissection (D2 and D3) has been routinely carried out in our department over the years and also in other institutions in Japan. The rate of D2 and D3 was as high as 89.3% for group 6. Therefore, curative resection (curability A and B) was consistently performed throughout.

© 2000 Cancer Research Campaign

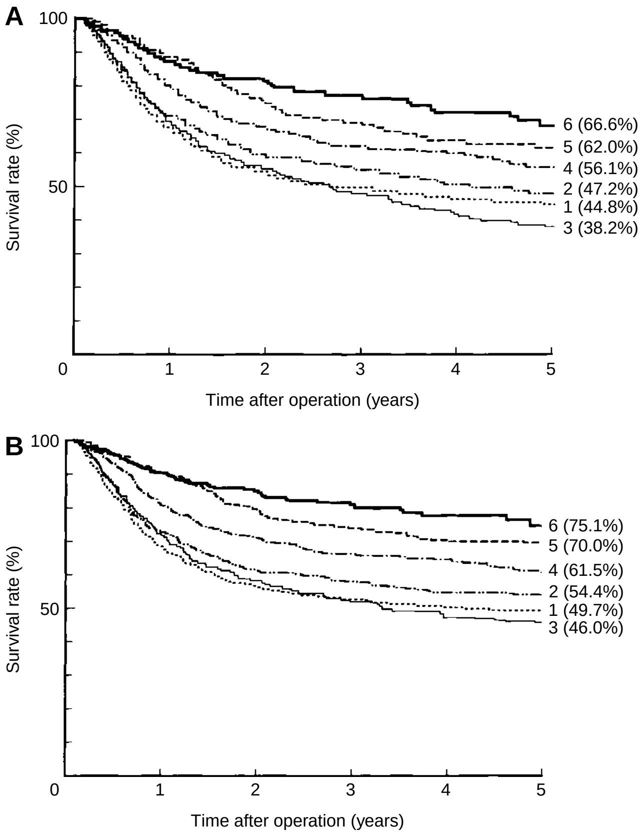

**Figure 2** Survival curves of patients undergoing surgery for gastric cancer. Differences in survival are significant (*P* = 0.0001) for mortality from gastric cancer and other diseases (**A**), and gastric cancer-related deaths only (**B**), among six groups. Five-year survival rates for all groups are indicated. Groups 1–6 were the same as in Figure 1

### Survival rates

Twenty-seven of 2152 patients (1.3%) died within the first 30 post-operative days; 2.4% (12/503) in group 1; 2.0% (7/350) in group 2; 1.2% (4/333) in group 3; 1.2% (4/325) in group 4; 0% in both groups 5 and 6. The rate of operative mortality has decreased in recent periods.

At the time these data were analysed, the median follow-up time for all 736 survivors was 9.4 years (0.1–32.5 years); 29.0 years (12.3–32.5) for 63 survivors in group 1; 23.6 years (4.4–26.5) for 70 survivors in group 2; 19.1 years (1.9–21.5) for 73 survivors in group 3; 13.3 years (0.3–16.5) for 124 survivors in group 4; 8.3 years (0.1–11.5) for 159 survivors in group 5; 4.0 years (0.1–6.8) for 247 survivors in group 6.

The survival rate of patients with gastric cancer was compared among the six groups (Figure 2). When gastric cancer deaths and non-gastric cancer deaths were all included in the survival analysis, the survival rate improved in recent periods (Figure 2A). The 5-year survival rate was 44.8% for group 1 and 66.6% for group 6. When non-gastric cancer deaths were considered as censored data, the disease-specific survival rate also improved in recent periods (Figure 2B). The 5-year survival rate was 49.7% for group 1 and 75.1% for group 6.

**Table 2** Cox regression analysis of data on patients with gastric cancer

| Explanatory variable (observed value) | *P* value | Relative risk (95% confidence interval) |
|---|---|---|
| Serosal invasion* (negative, positive) | 0.0001 | 2.5479 (2.0450–3.1747) |
| Lymph node metastasis (negative, positive) | 0.0001 | 3.1736 (2.4311–4.1432) |
| Lymph node dissection (D0&D1, D2&D3) | 0.0001 | 0.4621 (0.3709–0.5758) |
| Tumour size (per cm) | 0.0001 | 1.0775 (1.0534–1.1021) |
| Liver metastasis (negative, positive) | 0.0001 | 3.3877 (2.6158–4.3869) |
| Peritoneal dissemination (negative, positive) | 0.0001 | 1.6755 (1.6381–1.7137) |
| Lymphatic invasion (negative, positive) | 0.0053 | 1.3393 (1.1069–1.6203) |
| Vascular invasion (negative, positive) | 0.0069 | 1.2857 (0.9849–1.6784) |
| Location of the tumour (not whole stomach, whole stomach) | 0.0114 | 1.4039 (1.0840–1.8180) |
| Location of the tumour (middle stomach, others) | 0.0391 | 0.8201 (0.6779–0.9922) |

*Serosal invasion negative: m, sm, mp, ss; positive: se, si

### Multivariate analysis

To determine which of the covariates listed in Table 1 were pertinent prognostic factors of survival time for patients with gastric cancer, a multivariate analysis with Cox regression analysis with forward stepwise model was done for 1644 cases and all factors listed in Table 1 were identified. Ten factors of depth of invasion, lymph node metastasis, lymph node dissection, tumour size, liver metastasis, peritoneal dissemination, lymphatic invasion, vascular invasion, lesion in the whole stomach and lesion in the middle stomach were all independent prognostic factors (Table 2).

### DISCUSSION

This overview covering 31 years revealed that detection of gastric cancer at an early stage has increased with wide use of diagnostic procedures of radiography and endoscopy with targeted biopsy (Hisamichi, 1989). In Japan, mass screening also has a definite role in diagnosing gastric cancer, in its early stages (Abe et al, 1993). The high curative resectability rate in the screened group related to a smaller tumour size and to a lower incidence of lymph node metastasis, liver metastasis and peritoneal dissemination than in the the non-screened group. In the recent period, 57% of the tumours was early gastric cancer, in particular 34% was mucosal gastric cancer, and such screening has contributed to the recent decline in the number of deaths in subjects with this cancer. The definition of early gastric cancer was made by the Japanese Endoscopic Society in 1962 (Yoshimori, 1989), when endoscopic and radiologic examination were the only diagnostic tools used.

Metastasis to lymph nodes in directly linked to the outcome and extensive lymph node dissection is a statistically favourable prognostic factor (Maehara et al, 1991). In our study, 89.3% of the patients were treated with D2 or D3 lymph node disection. (Maehara et al, 1992). Baba et al (1995) reported that the rate of recurrence was higher in patients treated with dissection of group 1 lymph nodes than for those with dissection of group 2 or 3 nodes, in cases of a node-negative early gastric cancer. We noted

© 2000 Cancer Research Campaign cytokeratin-positive cancer cells in lymph nodes in 24% of the patients with node-negative early gastric cancer and who died with a recurrence (Maehara et al, 1996c). Siewert et al (1996) also reported that the presence of three or more tumour cells in more than 10% of the lymph nodes was of significant prognostic value in the pN0 cases.

Bonenkamp et al (1999) and Cuschieri et al (1999) reported that D2 patients had a higher operative mortality rate than did D1 patients and that these D2 patients experienced more complications, thus, D2 provided no survival benefit. Other workers reported that extensive lymph node dissection did not increase postoperative mortality rates, but did increase the morbidity rate (Wu et al, 1995; Robertson et al, 1994). Pacelli et al (1993) and de Manzoni et al (1996) found that extended lymphadenectomy significantly lengthened survival time for patients with gastric cancer, without increasing operative mortality. A prospective study done in Germany showed a significant survival advantage of radical lymphadenectomy for patients with stage II and stage IIIa disease (Siewert et al, 1993). Thus, there is controversy regarding the effects of lymph node dissection for gastric cancer (Bunt et al, 1994; Guadagni et al, 1995; McCulloch, 1995; Sakamoto and Yasue, 1995; Sue-Ling and Johnston, 1995). A number of factors contribute to the results of these clinical trials; such as surgeon's skill and personal attitude towards lymph node dissection, medical and nursing care, patient's constitution, the degree of obesity and multi-organ functions.

Roukos et al (1990) reported that 5.4% of the patients had metastases in group 2 nodes while group 1 nodes were unaffected. In these cases, the extent of lymph node metastasis was underestimated by D1 dissection. Therefore, D2 prophylactic lymph node dissection can make for a curative resection when micrometastases are present in group 2 lymph nodes. The remaining metastatic lesions in the lymph nodes may well explain the recurrence in a variety of organs, including liver and/or peritoneum (Maehara et al, 1995b). Preoperative diagnostic rate of lymph node metastasis was 37% in cases of a metastatic lymph node less than 15 mm, determined using helical CT (Fukuya et al, 1995). With respect to macroscopic findings at the time of operation, the correct diagnostic rate was only 15% in cases of metastatic lymph nodes less than 15 mm (Okamura et al, 1988). Thus, preoperative and macroscopic diagnosis will not precisely predict the presence of lymph node metastasis, and an exact statistical randomization of the patients to analyse effects of lymph node dissection against lymph node metastasis is difficult to carry out, in a prospective study. Prophylactic lymph node dissection will more likely lead to a curative procedure with no increase in operative morbidity (Maehara et al, 1997).

Patients with early gastric cancer with a smaller, macroscopically elevated and differentiated tumour can be treated by endoscopic resection (Sano et al, 1992). Lesions that can be endoscopically resected comprise elevated mucosal gastric cancer of less than 2 cm in diameter, and a depressed cancer of less than 1 cm. Laparoscopic-assisted gastric resection has been done (Kitano et al, 1995) and lymph node dissection was done safely using this procedure. Identification of sentinel lymph nodes by means of vital staining dye and/or lymphoscintingraphy (Alazraki et al, 1997) may facilitate a less invasive lymphadenectomy, however the reliability of these approaches for human gastric cancer should be further investigated.

The postoperative mortality was 2.4% (12/503) and 0% (0/348) for the earliest and latest period, respectively. Mass screening provides an excellent opportunity to detect gastric cancer in symptomless subjects (Hanazaki et al, 1997). Thus, the high percentage of such patients identified at an early stage will reduce mortality due to gastric cancer. The large decrease in operative mortality is due to improved surgical techniques but also to improvements in anaesthesis, metabolic care and intravenous nutrition (Sue-Ling et al, 1993).

Recurrences are likely to take on a variety of forms and in different organs, even after curative resection (Maehara et al, 1996b). Distant micrometastasis was detected in patients with gastric cancer (Schlimok et al, 1991; Maehara et al, 1996ac, 1998), and a number of factors are involved in these biological behaviours of gastric cancer (Maehara et al, 1999). In our institution, an earlier diagnosis, standardization of surgical techniques, including routinely performed lymph node dissection, close follow-up schedules for recurrences and perioperative managements for patients with gastric cancer have resulted in better survival rates after gastrectomy, in particular in the later part of the series.

### ACKNOWLEDGEMENTS

This work was supported by a Grant-in-Aid for Scientific Research from the Ministry of Education, Science, Sports and Culture of Japan.

### REFERENCES

Abe S, Lightdale CJ and Brennan MF (1993) The Japanese experience with endoscopic ultrasonography in the staging of gastric cancer. *Gastrointest Endosc* **39**: 586–591

Alazraki NP, Eshima D, Eshima LA, Herda SC, Murray DR, Vansant JP and Taylor AT (1997) Lymphoscintigraphy, the sentinel node concept, and the intraoperative gamma probe in melanoma, breast cancer and other potential cancers. *Semin Nucl Med* **27**: 55–67

Baba H, Maehara Y, Takeuchi H, Inutsuka S, Okuyama T, Adachi Y, Akazawa K and Sugimachi K (1995) Effect of lymph node dissection on the prognosis in patients with node-negative early gastric cancer. *Surgery* **117**: 165–169

Bonenkamp JJ, Hermans J, Sasako M, van de Velde, CJH and the Dutch Gastric Cancer Group (1999) Extended lymph-node dissection for gastric cancer. *N Engl J Med* **340**: 908–914

Bunt AMG, Hermans J, Boon MC, van de Velde CJH, Sasako M, Fleuren GJ and Bruijn JA (1994) Evaluation of the extent of lymphadenectomy in a randomized trial of Western- versus Japanese-type surgery in gastric cancer. *J Clin Oncol* **12**: 417–422

Cox DR (1972) Regression models and life tables. *J Royal Statist Soc Series B* **34**: 187–220

Cuschieri A, Weeden S, Fielding J, Bancewicz J, Craven J, Joypaul V, Sydes M, Fayers P, for the Surgical Co-operative Group (1999) Patient survival after D1 and D2 resections for gastric cancer: long-term results of the MRC randomized surgical trial. *Br J Cancer* **79**: 1522–1530

de Manzoni G, Verlato G, Guglielmi A, Laterza E, Genna M and Cordiano C (1996) Prognostic significance of lymph node dissection in gastric cancer. *Br J Surg* **83**: 1604–1607

Dixon WJ (1988) *BMDP Statistical Software*. Berkeley: University of California Press

Fuch CS and Mayer RJ (1995) Gastric carcinoma. *New Engl J Med* **333**: 32–41

Fukuya T, Honda H, Hayashi T, Kaneko K, Tateshi Y, Ro T, Maehara Y, Tanaka M, Tsuneyoshi M, Masuda K (1995) Lymph-node metastases: Efficacy of detection with helical CT in patients with gastric cancer. *Radiology* **197**: 705–711

Guadagni S, Catarci M and de Manzoni G (1995) D1 versus D2 dissection for gastric cancer. *Lancet* **345**: 1517

Hanazaki K, Sodeyama H, Wakabayashi M, Miyazaki M, Yokoyama S, Sode Y, Kawamura N, Miyazaki T and Ohtsuka M (1997) Surgical treatment of gastric cancer detected by mass screening. *Hepato-gastroenterol* **44**: 1126–1132

Hisamichi S (1989) Screening for gastric cancer. *World J Surg* **13**: 31–7

Japanese Gastric Cancer Association (1998) Japanese classification of gastric carcinoma -2nd English edition-. *Gastric Cancer* **1**: 10–24

 © 2000 Cancer Research Campaign

Jentschura D, Heubner C, Manegold BC, Rumstadt B, Winkler M and Trede M (1997) Surgery for early gastric cancer: A European one-center experience. *World J Surg* **21**: 845–849

Kaneko E, Nakamura T, Umeda N, Fujino M and Niwa H (1977) Outcome of gastric carcinoma detected by gastric mass survey in Japan. *Gut* **18**: 626–630

Kawasaki S (1975) A clinicopathological study on upward intramural extension of cancer of the stomach. *Fukuoka Acta Medica* **66**: 1–23 (in Japanese with English Abstract)

Kitano S, Shimoda K, Miyahara M, Shiraishi N, Bandoh T, Yoshida T, Shuto K and Kobayashi M (1995) Laparoscopic approaches in the management of patients with early gastric carcinomas. *Surg Laparosc Endosc* **5**: 359–362

Kodama Y, Sugimachi K, Soejima K, Matsusaka T and Inokuchi K (1981) Evaluation of extensive lymph node dissection for carcinoma of the stomach. *World J Surg* **5**: 241–248

Korenaga D, Okamura T, Baba H, Saito A and Sugimachi K (1988) Results of resection of gastric cancer extending to adjacent organs. *Br J Surg* **75**: 12–15

Maehara Y, Moriguchi S, Kakeji Y, Orita H, Haraguchi M, Korenaga D and Sugimachi K (1991) Prognostic factors in adenocarcinoma in the upper one-third of the stomach. *Surg Gynecol Obstet* **173**: 223–226

Maehara Y, Okuyama T, Moriguchi S, Orita H, Kusumoto H, Korenaga D and Sugimachi K (1992) Prophylactic lymph node dissection in patients with advanced gastric cancer promotes increased survival time. *Cancer* **70**: 392–395

Maehara Y, Okuyama T, Oshiro T, Baba H, Anai H, Akazawa K and Sugimachi K (1993) Early carcinoma of the stomach. *Surg Gynecol Obstet* **177**: 593–597

Maehara Y, Oshiro T, Oiwa H, Oda S, Baba H, Akazawa K and Sugimachi K (1995a) Gastric cancer in patients over 70 years of age. *Br J Surg* **82**: 102–105

Maehara Y, Oshiro T, Baba H, Ohno S, Kohnoe S and Sugimachi K (1995b) Lymphatic invasion and potential for tumor growth and metastasis in patients with gastric cancer *Surgery* **117**: 380–385

Maehara Y, Yamamoto M, Oda S, Baba H, Kusumoto T, Ohno S, Ichiyoshi Y and Sugimachi K (1996a) Cytokeratin-positive cells in bone marrow for identifying distant micrometastasis of gastric cancer. *Br J Cancer* **73**: 83–87

Maehara Y, Emi Y, Baba H, Adachi Y, Akazawa K, Ichiyoshi Y and Sugimachi K (1996b) Recurrences and related characteristics of gastric cancer. *Br J Cancer* **74**: 975–979

Maehara Y, Oshiro T, Endo K, Baba H, Oda S, Ichiyoshi Y, Kohnoe S and Sugimachi K (1996c) Clinical significance of occult micrometastasis in lymph nodes from patients with early gastric cancer who died of recurrence. *Surgery* **119**: 397–402

Maehara Y, Tomoda M, Tomisaki S, Ohmori M, Baba H, Akazawa K and Sugimachi K (1997) Surgical treatment and outcome for node-negative gastric cancer. *Surgery* **121**: 633–639

Maehara Y, Hasuda S, Abe T, Oki E, Kakeji Y, Ohno S and Sugimachi K (1998) Tumor angiogenesis and micrometastasis in bone marrow of patients with early gastric cancer. *Clin Cancer Res* **4**: 2129–2134

Maehara Y, Kakeji Y, Kabashima A, Emi Y, Watanabe A, Akazawa K, Baba H, Kohnoe S and Sugimachi K (1999) Role of transforming growth factor-β1 in invasion and metastasis in gastric carcinoma. *J Clin Oncol* **17**: 607–614

McCulloch P (1995) D1 versus D2 dissection for gastric cancer. *Lancet* **345**: 1516–1517

Moreaux J and Bougaran J (1993) Early gastric cancer. A 25-year surgical experience. *Ann Surg* **217**: 347–355

Noguchi Y, Imada T, Matsumoto A, Coit DG and Brennan MF (1989) Radical surgery for gastric cancer. A review of the Japanese experience. *Cancer* **64**: 2053–2062

Okamura T, Tsujitani S, Korenaga D, Haraguchi M, Baba H, Hiramoto Y and Sugimachi K (1988) Lymphadenectomy for cure in patients with early gastric cancer and lymph node metastasis. *Am J Surg* **155**: 476–480

Pacelli F, Doglietto GB, Bellantone R, Alfieri S, Sgadari A and Crucitti F (1993) Extensive versus limited lymph node dissection for gastric cancer: a comparative study of 320 patients. *Br J Surg* **80**: 1153–1156

Robertson CS, Chung SCS, Woods SDS, Griffin SM, Raimes SA, Lau JTF and Li AKC (1994) A prospective randomized trial comparing R1 subtotal gastrectomy with R3 total gastrectomy for antral cancer. *Ann Surg* **220**: 176–182

Roukos DH, Hottenrott C, Lorenz M and Koutsogiorgas-Couchell S (1990) A critical evaluation of effectivity of extended lymphadenectomy in patients with carcinoma of the stomach. *J Cancer Res Clin Oncol* **116**: 307–313

Sakamoto J and Yasue M (1995) Extensive lymphadenectomy for gastric cancer patients: what can the results of one trial tell us? *Lancet* **345**: 742–743

Sano T, Kobori O and Muto T (1992) Lymph node metastasis from early gastric cancer: endoscopic resection of tumour. *Br J Surg* **79**: 241–244

Schlimok G, Funke I, Pantel K, Strobel F, Lindemann F, Witte J and Riethmüller G (1991) Micrometastatic tumour cells in bone marrow of patients with gastric cancer: Methodological aspects of detection and prognostic significance. *Eur J Cancer* **27**: 1461–1465

Siewert JR, Böttcher K, Roder JD, Busch R, Hermanek P, Meyer HJ and the German Gastric Carcinoma Study Group (1993) Prognostic relevance of systematic lymph node dissection in gastric carcinoma. *Br J Surg* **80**: 1015–1018

Siewert JR, Kestlmeier R, Busch R, Böttcher K, Roder JD, Müller J, Fellbaum C and Höfler H (1996) Benefits of D2 lymph node dissection for patients with gastric cancer and pN0 and pN1 lymph node metastases. *Br J Surg* **83**: 1144–1147

Sue-Ling HM and Johnston D (1995) D1 versus D2 dissection for gastric cancer. *Lancet* **345**: 1515–1516

Sue-Ling HM, Johnston D, Martin IG, Dixon MF, Lansdown MRJ, McMahon MJ and Axon ATR (1993) Gastric cancer: a curable disease in Britain. *Gut* **307**: 591–596

Wu C-W, Hsieh M-C, Lo S-S, Wang L-S, Hsu W-H, Lui W-Y, Huang M-H and P'eng F-K (1995) Morbidity and mortality after radical gastrectomy for patients with carcinoma of the stomach. *J Am Coll Surg* **181**: 26–32

Yoshimori M (1989) The natural history of early gastric cancer. *Jpn J Clin Oncol* **19**: 89–93

© 2000 Cancer Research Campaign
